# Building radiomics models based on ACR TI-RADS combining clinical features for discriminating benign and malignant thyroid nodules

**DOI:** 10.3389/fendo.2025.1486920

**Published:** 2025-07-21

**Authors:** Xingxing Chen, Lili Zhang, Bin Chen, Jiajia Lu

**Affiliations:** ^1^ Department of Ultrasound, The First People’s Hospital of Xiaoshan District, Hangzhou, Zhejiang, China; ^2^ Clinical Research Center, Xiaoshan Affiliated Hospital of Wenzhou Medical University, Hangzhou, Zhejiang, China

**Keywords:** radiomics, ACR TI-RADS, thyroid nodules, nomogram, prediction

## Abstract

**Purpose:**

The aim of this study was to establish and validate a radiomics model combining the American College of Radiology Thyroid Imaging, Reporting and Data System (ACR TI-RADS) and clinical features and to build a nomogram that could be utilized to enhance the diagnostic performance of malignant thyroid nodules.

**Method:**

From January 2019 to September 2022, 329 thyroid nodules from 323 patients who had been referred for surgery and had pathological evidence of them were gathered retrospectively and randomly allocated to training and test cohorts (8:2 ratio). A total of 107 radiomics features were extracted from the US images, and the radiomics score (Rad-score) was constructed using the Least Absolute Shrinkage and Selection Operator (LASSO) algorithm. Different models were created using logistic regression, including the clinic-ACR score (Clin+ACR), clinic-Rad score (Clin+Rad), ACR score-Rad score (ACR+Rad), and combined clinic-ACR score-Rad score (Clin+ACR+Rad). The diagnostic performance of different models was calculated and compared using the area under the receiver operating curve (AUC) and the corresponding sensitivity and specificity.

**Results:**

Eight radiomics features were independent signatures for predicting malignant TNs, with malignant TNs having higher Rad-scores in both cohorts (*P* < 0.05). The Clin+ACR+Rad model showed excellent diagnostic prediction ability in both the training (AUC = 0.958) and test datasets (AUC = 0.937), significantly outperforming other models including Rad-score (AUC = 0.890, 0.856), Clin+Rad (AUC = 0.895, 0.859), ACR+Rad (AUC = 0.943, 0.934), and Clin+ACR (AUC = 0.784, 0.785) (all *P* < 0.05). The calibration curve demonstrated that the mean absolute error in the training group was just 0.020 and in the test cohort was 0.033. To evaluate the clinical utility of the nomogram in reducing unnecessary biopsies, we further analyzed the performance of our integrated model (Clin+ACR+Rad) compared to the traditional ACR TI-RADS system at different probability thresholds. At the statistically optimal threshold of 0.386, the unnecessary biopsy rate decreased from 46.97% to 22.05% in the training cohort and from 45.83% to 21.05% in the test cohort.

**Conclusion:**

The current study offers preliminary support that the model of combined clinic-ACR score-radiomics score can be helpful for predicting malignancy in thyroid nodules by looking at a retrospective cohort of surgically treated thyroid nodules. The Clin-ACR-Rad nomogram may be a more practical instrument and more accurate prediction model for malignant thyroid nodules.

## Introduction

Thyroid nodules (TNs) are a common disease of the endocrine system worldwide (1). The identification rate of TNs is increasing annually because of increased public health awareness and improved examination methods (2). But at the same time, the management of these nodules has significant clinical problems due to overdiagnosis and overtreatment (3). The pathological state of TNs is primarily related to patient prognosis and clinical management.

Currently, the pathological evaluation of TNs is primarily determined through fine-needle aspiration (FNA), an invasive procedure with inherent limitations, such as sampling errors and uncertain results (4). To minimize unnecessary invasive procedures, various non-invasive, ultrasound-based risk stratification systems have been developed. Among these, the American College of Radiology’s Thyroid Imaging Reporting and Data System (ACR TI-RADS) is the most widely used clinical tool. ACR TI-RADS assesses TNs based on five ultrasound features: composition, echogenicity, shape, margin, and echogenic foci (5, 6).

Despite proven efficacy in diagnosing TNs and reducing referral biopsies (7–9), ACR TI-RADS has notable limitations. The system may misclassify nodules with inconsistent ultrasound features (10), and relies on subjective visual interpretation, introducing inter-observer variability. To address these limitations, radiomics has emerged as a promising complementary approach. Radiomics enables high-throughput extraction of quantitative image features, capturing important aspects of the images, including histogram-based metrics and texture elements, which cannot be assessed by visual interpretation alone (11, 12). These extracted features may contain pathophysiological information related to the histological characteristics of tissues. Recent studies have shown that radiomic features from medical images are significantly associated with the histological staging of various diseases (11, 13, 14).

However, radiomics presents its own challenges. Radiological features are typically extracted from single 2D images of the target nodule, potentially overlooking important 3D characteristics (15). Moreover, focusing solely on imaging data disregards valuable clinical information that could enhance diagnostic accuracy. This suggests that combining radiomics with clinical data and standardized ultrasound evaluation systems may lead to better results. To address the limitations of individual methods and leverage their complementary strengths, we propose an integrated predictive model implemented through a nomogram.

A nomogram is a graphical tool that uses multivariate regression analysis to present predicted values for specific outcomes in a clear and interpretable manner. By integrating radiomic features (which capture subtle tissue patterns), ACR TI-RADS assessments (which provide a standardized evaluation of visible ultrasound features), and relevant clinical data (including patient-specific risk factors), we hypothesize that this combined approach will offer superior diagnostic performance compared to any single method.

Previous studies have attempted to combine radiomics with ACR TI-RADS to improve diagnostic accuracy, showing improved performance over single-modality approaches (16). Our study builds upon and extends this previous work by proposing a novel triple-modality approach (Clinical+ACR TI-RADS+Radiomics) that leverages the complementary strengths of all three feature sets. Therefore, the specific objectives of this study are: (1) to extract and select the optimal radiomic features from ultrasound images of thyroid nodules; (2) to develop and validate predictive models based on various combinations of radiomic features, ACR TI-RADS assessments, and clinical characteristics; (3) to evaluate and compare the diagnostic performance of different models for malignant thyroid nodules; and (4) to construct a comprehensive nomogram integrating these three dimensions.

## Materials and methods

The ethics committee of the local hospital approved this retrospective study (Ethical Approval NO. 2022-112). Patient confidentiality is strictly protected and the informed consent requirement was exempted. The research followed the guidelines set forth in the 1964 Helsinki Declaration and its revisions. The reporting of our radiomics study adheres to the CheckList for EvaluAtion of Radiomics research (CLEAR) guidelines (17). The completed CLEAR checklist is provided as a **Supplementary Material**.

### Patient enrollment

Consecutive patients who underwent thyroid ultrasonography and TNs found in our hospital between January 2019 and September 2022 were included. The nodules were subjected to the following inclusion and exclusion criteria.

The following were the inclusion criteria: (1) postoperative pathological findings were obtained following surgical excision of the target nodule; (2) The Philips iU22 system and an identical linear array transducer with a 5–12 MHz frequency bandwidth were used for the US assessment (Philips Ultrasound, Washington, USA); and (3) complete clinical data were obtained from medical records.

The exclusion criteria were categorized as follows: (1) TNs that produced controversial pathological outcomes; (2) unsatisfactory US image quality, which affected the feature extraction; and (3) patients who underwent other treatments such as chemotherapy, radiation, or radiofrequency ablation before surgery.

This study included 323 patients (91 males and 238 females; mean age 53.1 years, range 20–84 years), with a total of 329 thyroid nodules. Six patients had multiple nodules, which were randomly assigned during dataset splitting to ensure statistical independence. Of all nodules, 161 were malignant and 168 were benign. Among the 161 malignant nodules, 158 cases (98.1%) of papillary thyroid carcinoma, 2 cases (1.2%) of follicular thyroid carcinoma, and 1 case (0.6%) of medullary thyroid carcinoma. All 329 nodules were divided into two groups at random, with an 8:2 ratio: a training cohort (n = 263, 75 men and 188 women; median age, 53.6 years, range 20 to 83 years) and a test cohort (n = 66, 16 men and 50 women; median age, 51.2 years, range 22 to 84 years).

### Clinical and US information

Clinical data, including age, sex, body mass index (BMI), medical history, smoking, and alcohol drinking, were obtained from the medical records. The gold standard was established when expert pathologists validated pathological findings. In our investigation, identical Philips iU22 equipment and a linear array transducer were used for the US examinations. All US images were evaluated by two experienced radiologists (10 and 7 years), who were unaware of any clinical details or final pathological diagnoses. B-mode ultrasonography (BMUS) features of the TNs were collected using the ACR TI-RADS standard (18). Two radiologists estimated each nodule’s score and characterized its B-mode ultrasound parameters, such as echogenicity, composition, shape, margins, and echogenic foci. If there were any disagreements, the final diagnosis was based on consensus.

### TNs segmentation

From the image storage system, the TNs with the largest diameter in the B-mode ultrasound images were selected, and ITK-SNAP software was then used for analysis (version 3.6.0). A radiologist with 10 years of experience in thyroid ultrasound diagnosis manually delineated the region of interest (ROI) to define the boundaries of the TN. To enhance the visibility of the nodule’s borders, contrast and brightness settings in ITK-SNAP were adjusted, typically increasing the contrast by about 15% and reducing the brightness by approximately 10%. The largest cross-sectional image of each nodule was selected for ROI delineation. The ROI boundaries included not only the nodule itself but also the surrounding area with observable changes in echogenicity, which were assessed visually by the experienced radiologist.

To evaluate intra-observer reproducibility, the radiologist randomly selected 50 TNs and described them twice, with a two-week interval between assessments. Additionally, another radiologist, with 7 years of experience in thyroid ultrasound diagnosis, independently described the ROIs of the same 50 TNs to assess inter-observer reproducibility. Both radiologists were blinded to any clinical details or final pathological diagnoses. The reproducibility was quantitatively measured using intraclass correlation coefficient (ICC). The evaluation showed good reproducibility with an intra-observer ICC of 0.87 (95% CI: 0.82-0.92) and an inter-observer ICC of 0.82 (95% CI: 0.76-0.88).

### Radiomics feature extraction

We extracted 107 radiomics features using the open-source Python library “Pyradiomics V1.3.0” (http://www.radiomics.io/pyradiomics.html). For image preprocessing and feature extraction, the following parameters were used: (1) Image resampling was performed using B-spline interpolation method while maintaining the original image resolution; (2) Discretization utilized a fixed bin width method with a bin width of 25; (3) 2D features were extracted (force2D: true) from the segmented ROIs. We chose 2D rather than 3D feature extraction because clinical thyroid nodule assessment typically uses 2D ultrasound of the largest cross-sectional plane, 2D processing requires less computational resources, and alignment with clinical practice enhances result interpretability and clinical applicability; (4) A total of 107 radiomic features were extracted from seven feature classes, including first-order statistics features (n=18), gray-level co-occurrence matrix features (GLCM, n=24), gray-level run-length matrix features (GLRLM, n=16), gray level size zone matrix features (GLSZM, n=16), shape-based features (n=14), gray level dependence matrix features (GLDM, n=14), and neighboring gray tone difference matrix features (NGTDM, n=5); (5) All other parameters remained as default configurations in Pyradiomics V1.3.0. The extracted features followed the Image Biomarker Standardization Initiative (IBSI) guidelines to ensure reproducibility. We limited our analysis to these 107 features to maintain a reasonable feature-to-sample ratio (approximately 1:3) given our sample size (329 nodules), thereby reducing the risk of overfitting. Moreover, these features cover the most commonly used features in radiomic analysis and are particularly suitable for ultrasound image analysis, which typically has lower resolution and contrast compared to CT or MRI.

Normalization based on Z-score features was applied, where the mean of radiomic features was standardized to 0 and the standard deviation to 1. Features with an ICC greater than 0.75 were considered to have strong consistency and were included in the subsequent feature selection process.

### Feature selection and Rad-score establishment

**Figure 1:**
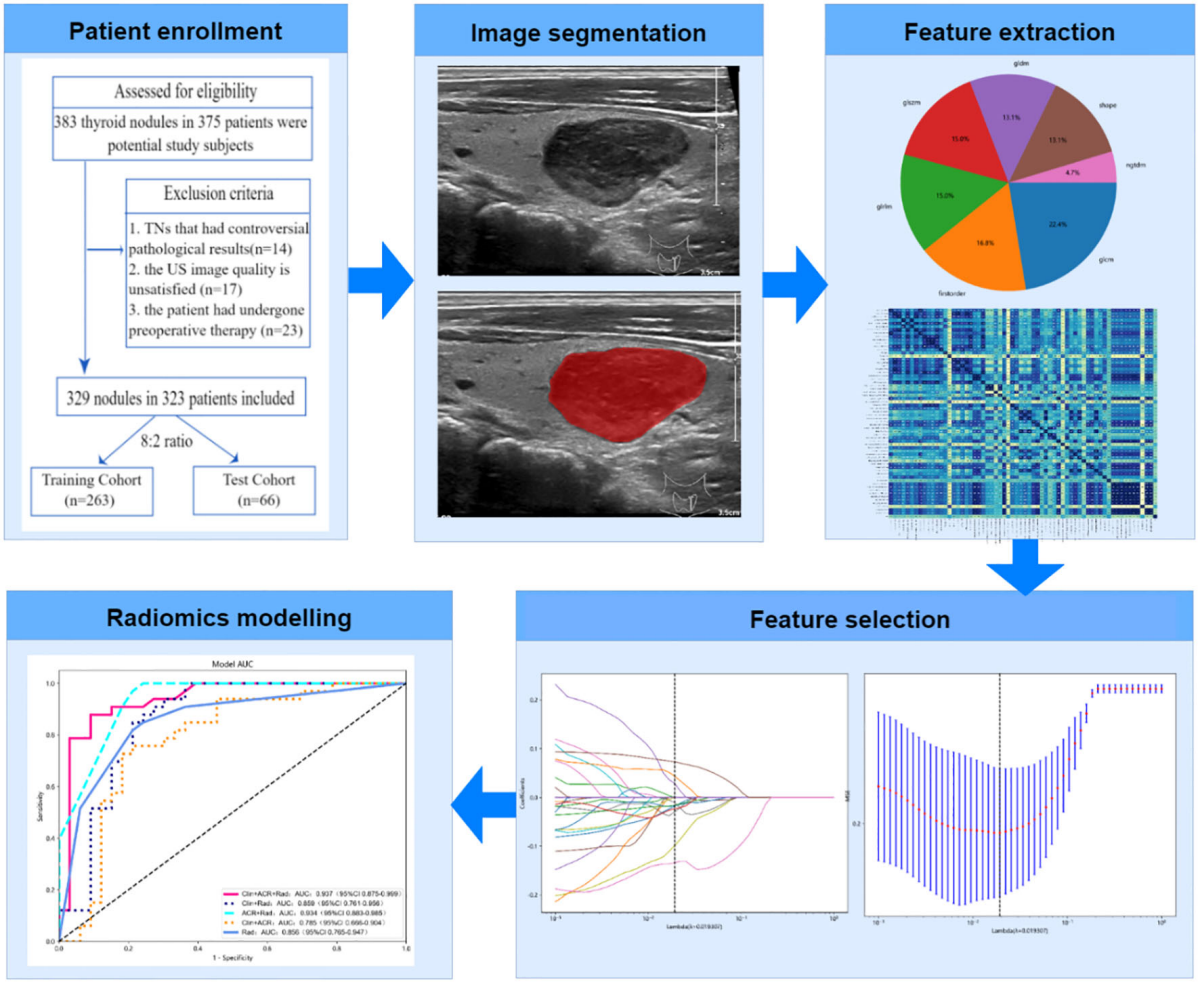
Flowchart of radiomics analysis process.

First, to identify the picture attributes with statistically significant differences(*P*<0.05), a two-sample t-test was used with the goal of removing unnecessary feature parameters to reduce the overfitting data dimension. To exclude picture characteristics with correlations less than 0.8, Spearman’s correlation analysis was performed. Finally, to identify the top-ranked features, the Least Absolute Shrinkage and Selection Operator (LASSO) logistic regression approach is employed by adjusting the penalty parameter using 5-fold cross-validation. A radiomics signature, also known as a radiomics score (Rad-score), was generated by weighting the chosen attributes with nonzero coefficients using the results of a linear regression model. **Figure 1** depicts the flowchart of the radiomics analysis procedure.

### Statistical analysis

Data were analyzed using R software (version 3.7.0) and SPSS software (version 22.0). Categorical data are reported as numbers and percentages, whereas continuous data are expressed as mean ± standard deviation. The student’s t-test was used to compare continuous data, and the chi-square test or, if appropriate, Fisher’s test was used to compare categorical data. Univariate logistic regression was used in the training cohort to examine factors that predicted malignancy. To assess the applicability of all putative predictors of malignancy, regression coefficients (β) and odds ratios (ORs) with corresponding relative 95% confidence intervals (CIs) were calculated.

Different models were created using logistic regression, taking into consideration the possible impact of clinical factors for each patient, including the clinic-ACR score (Clin+ACR), clinic-Rad score (Clin+Rad), ACR score-Rad score (ACR+Rad), and combined clinic-ACR score-Rad score (Clin+ACR+Rad). We chose logistic regression over other machine learning methods (such as random forest) because logistic regression models have stronger interpretability, making them easier for clinicians to understand and apply. Additionally, considering our relatively limited sample size (329 nodules), logistic regression is less prone to overfitting compared to more complex models. It was feasible to discriminate between benign and malignant TNs using the area under the receiver operating curve (AUC) and associated specificity, sensitivity, negative predictive value (NPV), and positive predictive value (PPV). High (AUC>0.9), moderate (AUC = 0.7-0.9), or low (AUC = 0.5-0.7) diagnostic performance was considered (19). The optimal cut-off values for each model were determined based on the Youden index (sensitivity + specificity - 1) in the ROC curve analysis. In addition, we compared the performance of all models to a zero-rule classifier (that is, a classifier that predicts all nodules as the most common category in the dataset) to assess how much our model improves against a simple benchmark. The nomogram was built based on the results, and calibration was evaluated using the calibration curve.

## Results

### Demographics and thyroid nodules characteristics

**Table 1** displays the baseline information of the recruited nodules in the training and test groups. The frequency of malignant lesions did not change significantly between the training and test groups [48.7% (128/263) vs. 50.0% (33/66), *P* = 0.847]. Additionally, there were no significant variations in age, sex, BMI, alcohol and tobacco use, medical history, or nodule diameter between the two cohorts.

**Table 1:** Information for thyroid nodules in the training and test cohorts.

Characteristics	Entire TNs (n = 329)	Training cohort (n = 263)	Test cohort (n = 66)	*P*-value
Nodule pathology				0.847
Benign	168 (51.1%)	135 (51.3%)	33 (50.0%)	
Malignant	161 (48.9%)	128 (48.7%)	33 (50.0%)	
Age (years)	53.1 ± 12.7	53.6 ± 12.4	51.2 ± 13.8	0.175
Gender				0.488
Male	238 (72.3%)	188 (71.5%)	50 (75.8%)	
Female	91 (27.7%)	75 (28.5%)	16 (24.2%)	
Diameter(cm)	1.7 ± 1.4	1.7 ± 1.4	1.7 ± 1.5	0.662
BMI	24.4 ± 3.8	24.5 ± 3.9	24.2 ± 3.4	0.600
Smoking				0.613
Yes	62 (18.8%)	51 (19.4%)	11 (16.7%)	
No	267 (81.2%)	212 (80.6%)	55 (83.3%)	
Alcohol drinking				0.613
Yes	62 (18.8%)	51 (19.4%)	11 (16.7%)	
No	267 (81.2%)	212 (80.6%)	55 (83.3%)	
Diabetes				0.813
Yes	42 (12.8%)	33 (12.5%)	9 (13.6%)	
No	287 (87.2%)	230 (87.5%)	57 (86.4%)	
Hypertension				0.088
Yes	98 (29.8%)	84 (31.9%)	14 (21.2%)	
No	231 (70.2%)	179 (68.1%)	52 (78.8%)	
Composition				0.049
Cystic	2 (0.6%)	2 (0.8%)	0 (0.0%)	
Spongiform	8 (2.4%)	4 (1.5%)	4 (6.1%)	
Cystic-solid mixture	63 (19.1%)	46 (17.5%)	17 (25.8%)	
Solid	256 (77.8%)	211 (80.2%)	45 (68.2%)	
Echogenicity				0.625
Anechoic	3 (0.9%)	3 (1.1%)	0 (0.0%)	
Hyper- or isoechoic	84 (25.5%)	70 (26.6%)	14 (21.2%)	
Hypoechoic	236 (71.7%)	185 (70.3%)	51 (77.3%)	
Very hypoechoic	6 (1.8%)	5 (1.9%)	1 (1.5%)	
Shape				0.721
Wider-than-tall	178 (54.1%)	141 (53.6%)	37 (56.1%)	
Taller-than-wide	151 (45.9%)	122 (46.4%)	29 (43.9%)	
Margins				0.176
Smooth	157 (47.7%)	118 (44.9%)	39 (59.1%)	
Blurry	69 (21.0%)	60 (22.8%)	9 (13.6%)	
Irregular or lobulated	89 (27.1%)	74 (28.1%)	15 (22.7%)	
Extrathyroidal extension	14 (4.3%)	11 (4.2%)	3 (4.5%)	
Echogenic foci				0.546
No calcification or with comet tail sign	170 (51.7%)	138 (52.5%)	32 (48.5%)	
Coarse calcification	51 (15.5%)	42 (16.0%)	9 (13.6%)	
Perinodular annular calcification	4 (1.2%)	4 (1.5%)	0 (0.0%)	
Microcalcification	91 (27.7%)	68 (25.9%)	23 (34.8%)	
More than two types of calcification	13 (4.0%)	11 (4.2%)	2 (3.0%)	
ACR-score	6.7 ± 3.2	6.7 ± 3.2	6.6 ± 3.4	0.867
Rad-score	0.5 ± 0.3	0.5 ± 0.3	0.5 ± 0.3	0.350

TNs, Thyroid Nodules; BMI, body mass index; ACR-score, ACR TI-RADS score; Rad-score, radiomics score.

*P*-values reflect differences between the training and test cohorts. The numbers in parentheses are percentages.

In the training and test cohorts, we also examined the fundamental information mentioned above in relation to malignant and benign nodules. **Table 2** presents this information. Age and nodule diameter were significantly different (all *P* < 0.05). In addition, between benign and malignant nodules, there were noticeable changes in the five ACR TI-RADS parameters and ACR scores. In both the training and test groups, malignant nodules had a substantially higher ACR-Score than benign nodules (*P* < 0.05).

**Table 2:** Information for thyroid nodules in the training and validation cohorts (stratified by pathology).

Characteristics	Training cohort		*P*-value	Test cohort		*P*-value
	Benign (n =135)	Malignant (n = 128)		Benign (n =33)	Malignant (n = 33)	
Age (years)	56.7 ± 10.5	50.4 ± 13.4	<0.001	52.7 ± 14.7	49.7 ± 12.7	0.038
Gender			0.494			1.000
Male	94 (69.6%)	94 (73.4%)		25 (75.8%)	25 (75.8%)	
Female	41 (30.4%)	34 (26.6%)		8 (24.2%)	8 (24.2%)	
Diameter(cm)	2.2 ± 1.7	1.1 ± 0.7	<0.001	2.6 ± 1.8	0.9 ± 0.5	<0.001
BMI	24.3 ± 3.2	24.7 ± 4.5	0.345	23.7 ± 2.9	24.7 ± 3.7	0.231
Smoking			0.956			0.741
Yes	109 (80.7%)	103 (80.5%)		28 (84.8%)	27 (81.8%)	
No	26 (19.3%)	25 (19.5%)		5 (15.2%)	6 (18.2%)	
Alcohol drinking			0.798			0.741
Yes	108 (80.0%)	104 (81.2%)		28 (84.8%)	27 (81.8%)	
No	27 (20.0%)	24 (18.8%)		5 (15.2%)	6 (18.2%)	
Diabetes			0.470			0.720
Yes	120 (88.9%)	110 (85.9%)		29 (87.9%)	28 (84.8%)	
No	15 (11.1%)	18 (14.1%)		4 (12.1%)	5 (15.2%)	
Hypertension			0.196			0.547
Yes	87 (64.4%)	92 (71.9%)		25 (75.8%)	27 (81.8%)	
No	48 (35.6%)	36 (28.1%)		8 (24.2%)	6 (18.2%)	
Composition			<0.001			<0.001
Cystic	2 (1.5%)	0 (0.0%)		0 (0.0%)	0 (0.0%)	
Spongiform	4 (3.0%)	0 (0.0%)		3 (9.1%)	1 (3.0%)	
Cystic-solid mixture	44 (32.6%)	2 (1.6%)		16 (48.5%)	1 (3.0%)	
Solid	85 (63.0%)	126 (98.4%)		14 (42.4%)	31 (93.9%)	
Echogenicity			<0.001			0.001
Anechoic	3 (2.2%)	0 (0.0%)		0 (0.0%)	0 (0.0%)	
Hyper- or isoechoic	61 (45.2%)	9 (7.0%)		13 (39.4%)	1 (3.0%)	
Hypoechoic	70 (51.9%)	115 (89.8%)		20 (60.6%)	31 (93.9%)	
Very hypoechoic	1 (0.7%)	4 (3.1%)		0 (0.0%)	1 (3.0%)	
Shape			<0.001			<0.001
Wider-than-tall	98 (72.6%)	43 (33.6%)		27 (81.8%)	10 (30.3%)	
Taller-than-wide	37 (27.4%)	85 (66.4%)		6 (18.2%)	23 (69.7%)	
Margins			<0.001			0.001
Smooth	92 (68.1%)	26 (20.3%)		27 (81.8%)	12 (36.4%)	
Blurry	24 (17.8%)	36 (28.1%)		1 (3.0%)	8 (24.2%)	
Irregular or lobulated	19 (14.1%)	55 (43.0%)		5 (15.2%)	10 (30.3%)	
Extrathyroidal extension	0 (0.0%)	11 (8.6%)		0 (0.0%)	3 (9.1%)	
Echogenic foci			<0.001			<0.001
No calcification or with comet tail sign	94 (69.6%)	44 (34.4%)		25 (75.8%)	7 (21.2%)	
Coarse calcification	21 (15.6%)	21 (16.4%)		6 (18.2%)	3 (9.1%)	
Perinodular annular calcification	3 (2.2%)	1 (0.8%)		0 (0.0%)	0 (0.0%)	
Microcalcification	15 (11.1%)	53 (41.4%)		2 (6.1%)	21 (63.6%)	
More than two types of calcification	2 (1.5%)	9 (7.0%)		0 (0.0%)	2 (6.1%)	
ACR-score	4.8 ± 2.8	8.7 ± 2.2	<0.001	4.2 ± 2.8	9.1 ± 1.8	<0.001
Rad-score	0.2 ± 0.1	0.8 ± 0.1	<0.001	0.3 ± 0.3	0.6 ± 0.2	<0.001

BMI, body mass index; ACR-score, ACR TI-RADS score; Rad-score, radiomics score.

*P*-values reflect differences between malignant and benign nodules. The numbers in parentheses are percentages.

### Feature selection and Rad-score calculation

**Figure 2:**
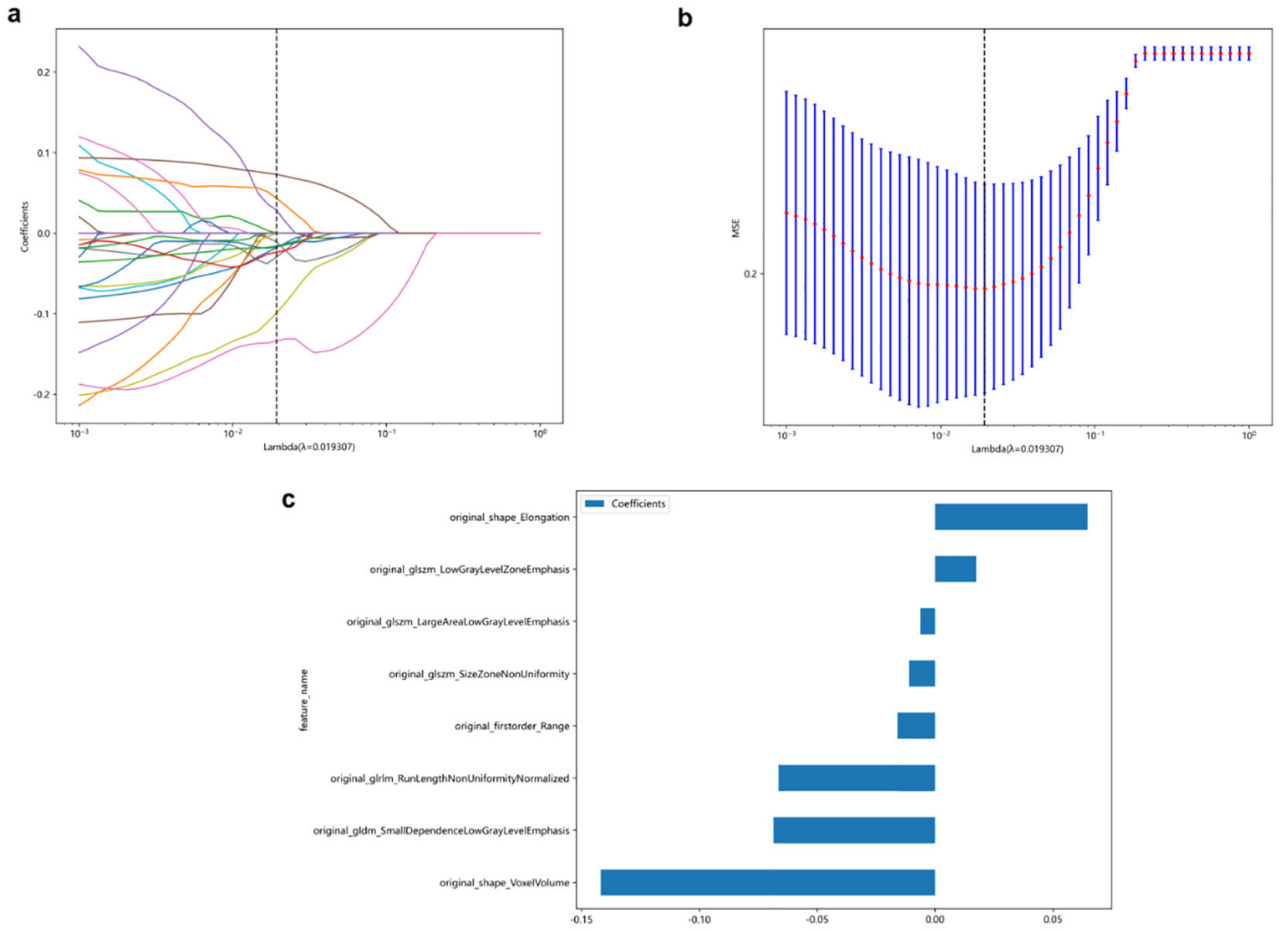
The least absolute shrinkage and selection operator (LASSO) logistic regression was used to identify the top-ranked features. **(a, b)** Five-fold cross-validation was used to select the tuning parameter (λ) in the LASSO regression model. An optimal λ value of 0.0193 was selected. **(c)** The eight selected features and coefficients of the Rad-score were indicated by the y-axis and x-axis, respectively.


First, to avoid overfitting, 107 extracted features were reduced to 27 features using t-test and Spearman analysis. The LASSO approach was then used for final dimensionality reduction and feature selection to generate the eight traits that were most useful in distinguishing between benign and malignant TNs. Based on these eight selected traits, we developed the following Rad-score calculation (Lambda[λ] = 0.0193, five-fold cross-validation) (**Figure 2**):

$$\begin{array}{c} Rad-score=0.499-0.015*original\_firstorder\_Range\\ -0.068*original\_gldm\_SmallDependenceLowGrayLevelEmphasis\\ -0.066*original\_glrlm\_RunLengthNonUniformityNormalized\\ -0.006*original\_glszm\_LargeAreaLowGrayLevelEmphasis\\ +0.017*original\_glszm\_LowGrayLevelZoneEmphasis\\ -0.011*original\_glszm\_SizeZoneNonUniformity\\ +0.0646*original\_shape\_Elongation\;-0.141\;\\ {}*\;original\_shape\_VoxelVolume \end{array}$$

The eight selected radiomics features in the Rad-score formula capture different aspects of thyroid nodules that are clinically relevant for malignancy prediction. Original_shape_Elongation quantifies the stretching or elongation of a nodule, with higher values indicating more irregular, elongated shapes. This aligns with the clinical ‘taller-than-wide’ sign, as malignant nodules tend to grow across normal tissue planes. Original_shape_VoxelVolume represents the three-dimensional size of the nodule, providing volumetric information beyond simple diameter measurements.

The textural features in our model characterize internal nodule heterogeneity that may not be visible to the naked eye. Original_firstorder_Range measures the variation in echogenicity within the nodule, reflecting internal structural complexity. Original_glszm_LowGrayLevelZoneEmphasis quantifies the presence of hypoechoic regions, commonly associated with malignancy, while original_glszm_LargeAreaLowGrayLevelEmphasis describes the distribution of larger hypoechoic areas that may represent cystic changes or necrosis. Original_glszm_SizeZoneNonUniformity evaluates the variability in the size of similarly echogenic regions, indicating structural heterogeneity. Original_gldm_SmallDependenceLowGrayLevelEmphasis identifies small, discrete hypoechoic areas that could represent microcalcifications or small areas of necrosis, and original_glrlm_RunLengthNonUniformityNormalized captures the complexity and discontinuity of echo texture patterns, reflecting the degree of tissue disorganization. Together, these quantitative features detect subtle malignancy-associated patterns beyond conventional visual assessment capabilities.

According to **Table 2**, whether in the training group (0.2 ± 0.1 vs. 0.8 ± 0.1, *P*<0.001) or test group (0.3 ± 0.3 vs. 0.6 ± 0.2, *P*<0.001), benign TNs had substantially lower Rad-scores than malignant TNs.

### Performance and validation of different models

**Figure 3:**
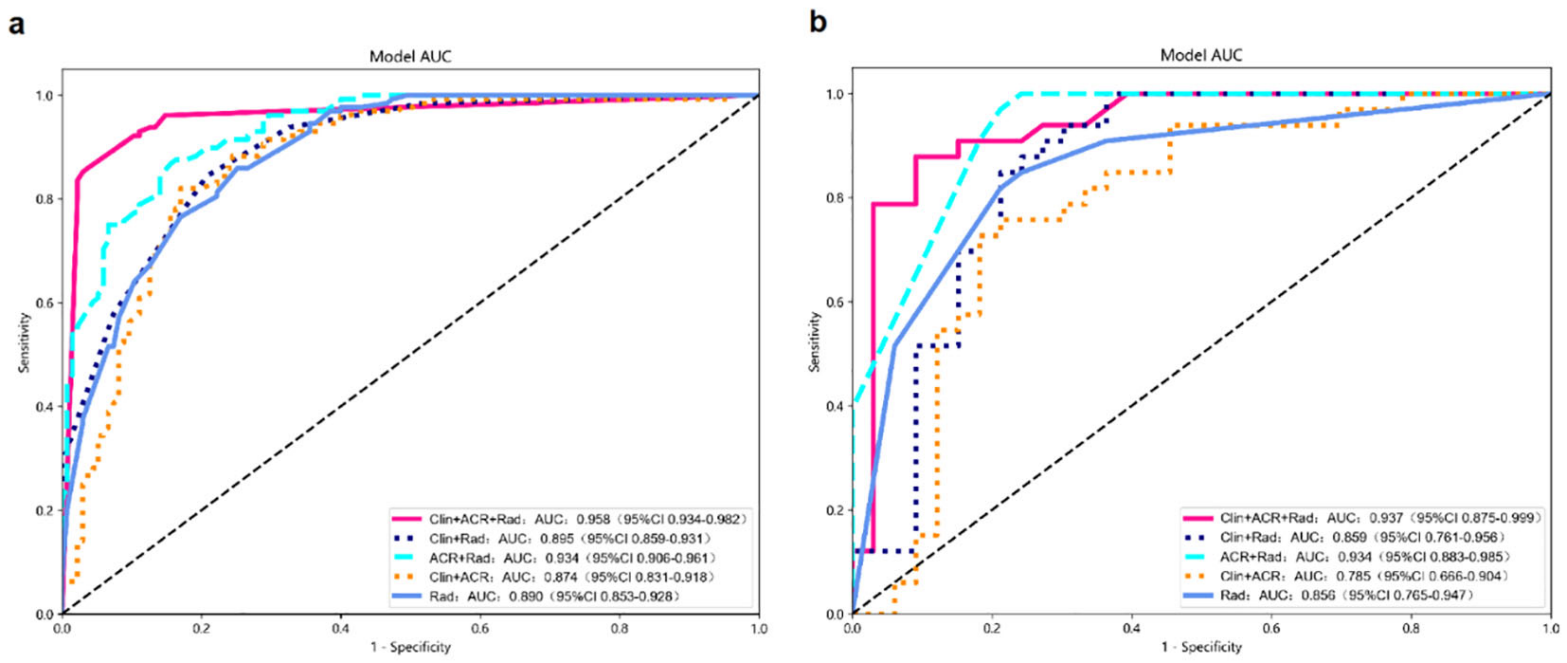
Diagnostic performance of different models for predicting malignant TNs. **(a)** AUC graphics of different models for predicting malignant TNs in the training cohort; **(b)** AUC graphics of different models for predicting malignant TNs in the test cohort.

In the training dataset, the Clin+ACR+Rad model based on the optimal cut-off value of 0.386 showed excellent diagnostic prediction ability, with sensitivity of 98.43%, specificity of 89.24%, and AUC value of 0.958, which was significantly superior to other models. It includes the Rad-score model with a cut-off of 0.485 (AUC = 0.890), the Clin+Rad model with a cut-off of 0.572 (AUC = 0.895), and the ACR+Rad model with a cut-off of 0.396 (AUC = 0.943) and a Clin+ACR model with a cut-off value of 0.628 (AUC = 0.784) (all *P* values < 0.05) (**Figure 3a**, **Table 3**). This indicates that the Clin+ACR+Rad model can effectively distinguish benign and malignant thyroid nodules with an optimal cut-off value of 0.386, and maintains stable high performance in independent test sets. Similarly, when compared with the Rad-score (AUC = 0.856), Clin+Rad (AUC = 0.859), ACR+Rad (AUC = 0.934), and Clin+ACR (AUC = 0.785), the AUC value for Clin+ACR+Rad in the test group was substantially higher (AUC = 0.937) (all *P* < 0.05) (**Figure 3b**, **Table 3**). In our dataset, 168 nodules were benign (51.1%) and 161 were malignant (48.9%). Using a zero-rule classifier (which predicts all nodules as benign, the most common class in the dataset), the accuracy would be 51.1%. In contrast, our Clin+ACR+Rad model achieved accuracies of 93.6% in the training set and 86.4% in the test set, significantly outperforming the zero-rule classifier. This demonstrates that our model offers substantial clinical value compared to the simplest predictive strategy.

**Table 3:** Diagnostic performances of models.

Models	Cut-off value	Training cohort					Test cohort				
		AUC (95% CI)	SEN(%)	SPE(%)	PPV(%)	NPV(%)	AUC (95% CI)	SEN(%)	SPE(%)	PPV(%)	NPV(%)
Clin+ACR+Rad	0.386	0.958 (0.941-0.975)	98.43	89.24	77.68	78.51	0.937 (0.907-0.967)	93.90	79.64	77.46	74.21
Clin+Rad	0.572	0.895^#^ (0.872-0.918)	82.81	68.88	71.62	80.86	0.859^##^ (0.820-0.898)	75.75	78.78	78.12	76.47
ACR+Rad	0.396	0.943^$^ (0.924-0.962)	89.21	87.77	81.69	79.24	0.934^$$^ (0.903-0.965)	84.84	75.75	77.78	83.33
Clin+ACR	0.628	0.784^&^ (0.750-0.818)	72.18	69.25	71.83	78.51	0.785^&&^ (0.736-0.834)	72.72	84.37	79.31	72.97
Rad	0.485	0.890^*^ (0.867-0.913)	93.93	79.37	79.44	73.33	0.856^**^ (0.816-0.896)	82.43	79.25	78.67	78.50

Clinical features: ACR, ACR TI-RADS score; Rad, radiomics score; AUC, area under receiver operating characteristics; CI, confidence interval; Sen, sensitivity; Spe, specificity; PPV, positive predictive value; NPV, negative predictive value.

^##^indicates a significant difference compared with that of Clin+ACR+Rad in the training cohort (*P* = 0.016).

^##^indicates a significant difference compared with that of Clin+ACR+Rad in the test cohort (*P* = 0.028).

^$^indicates a significant difference compared with that of Clin+ACR+Rad in the training cohort (*P* = 0.036).

^$$^indicates a significant difference compared to that of Clin+ACR+Rad in the test cohort (*P* = 0.045).

^&^indicates a significant difference compared with that of Clin+ACR+Rad in the training cohort (*P <*0.001).

^&&^indicates a significant difference compared with Clin+ACR+Rad in the test cohort (*P* = 0.008).

^*^indicates a significant difference compared with that of Clin+ACR+Rad in the training cohort (*P* = 0.021).

^**^indicates a significant difference compared with that of Clin+ACR+Rad in the test cohort (*P* = 0.036).

**Figure 4:**
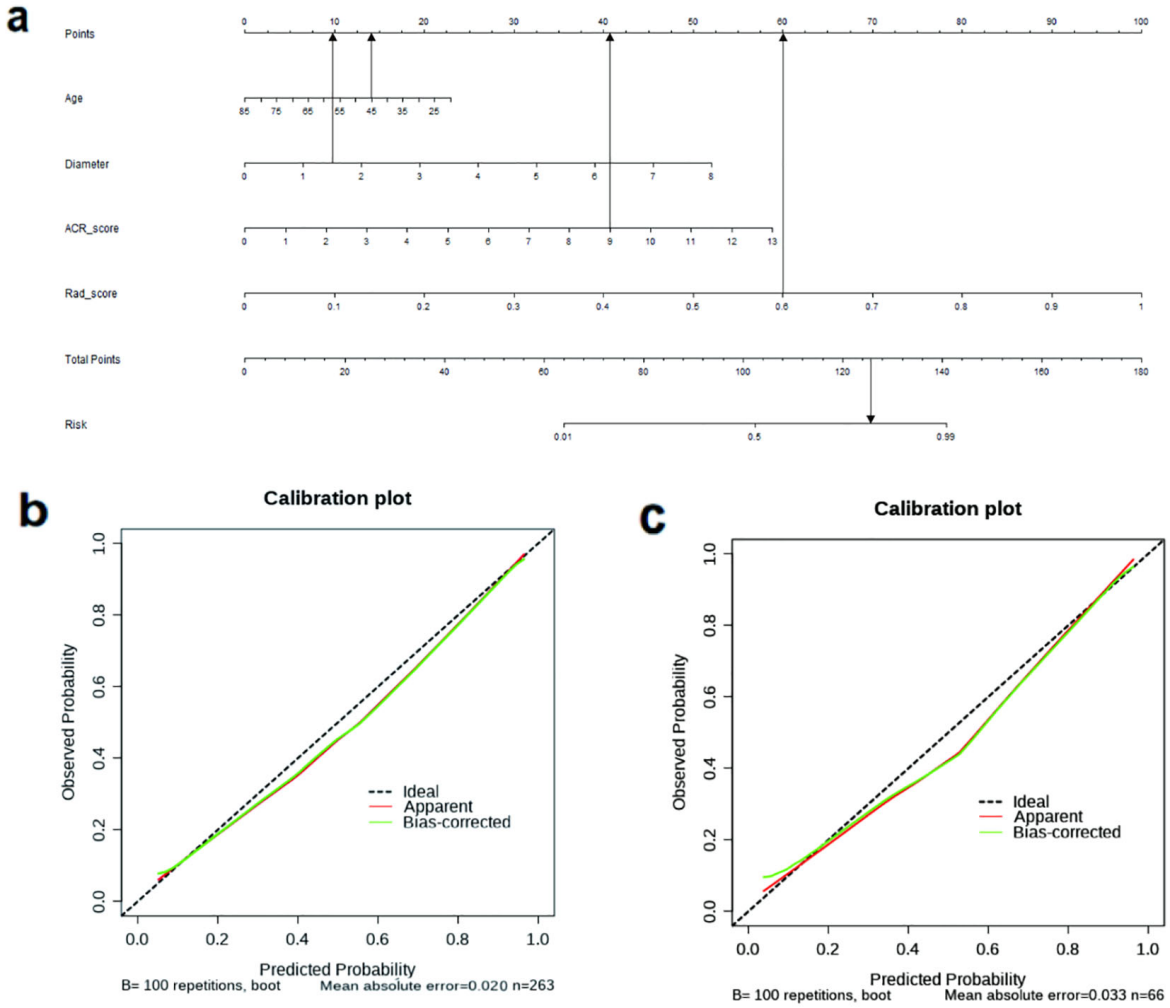
**(a)** The nomogram based on the model of combined clinical characteristics -ACR score-radiomics score. Usage instructions: (1) Locate patient values on each variable axis (Age, Diameter, ACR-Score, and Rad-score); (2) Draw vertical lines to the ‘Points’ axis to determine points for each variable; (3) Sum all points to obtain ‘Total Points’; (4) Draw a vertical line from the ‘Total Points’ axis down to the ‘Probability’ axis to determine the malignancy probability. **(b)** The calibration curve showed a mean absolute error of 0.020 in training cohort. result = 1: The pathological state of a nodule was malignant. **(c)** The calibration curve showed a mean absolute error of 0.033 in test cohort. result = 1: The pathological state of a nodule was malignant.

**Figure 4** displays the nomogram, which is based on the clinical characteristics, Rad-score, ACR-score, and calibration plot. To illustrate the clinical application of this nomogram, consider the following example (shown in **Figure 4a**): A 45-year-old patient presents with a thyroid nodule measuring 1.5 cm in diameter. Ultrasound examination gives an ACR TI-RADS score of 9, and the radiomics analysis yields a Rad-score of 0.6. Using the nomogram, the clinician first locates the patient’s age (45) on the Age axis and draws a line upward to the Points axis, obtaining approximately 14 points. Similarly, for nodule diameter (1.5 cm), ACR score (7), and Rad-score (0.6), the corresponding points are approximately 10, 41, and 60, respectively. The clinician then sums these values to get a total of 125 points. Locating this value on the Total Points axis and drawing a line downward to the Probability axis indicates approximately 85% probability of malignancy. Based on this high probability, the clinician would likely recommend fine-needle aspiration biopsy or surgical intervention rather than observation.

The calibration curve (**Figures 4b, c**) demonstrated that the mean absolute error in the training group was just 0.020 and in the test cohort was 0.033. To evaluate the clinical utility of the nomogram in reducing unnecessary biopsies, we further analyzed the performance of our integrated model (Clin+ACR+Rad) compared to the traditional ACR TI-RADS system at different probability thresholds. As shown in the **Supplementary Table 1**, at the statistically optimal threshold of 0.386, the unnecessary biopsy rate decreased from 46.97% to 22.05% in the training cohort and from 45.83% to 21.05% in the test cohort, while maintaining high sensitivity (98.43% and 93.90%, respectively).

The reduction in unnecessary biopsies may vary depending on clinical settings, patient populations, and the threshold selected by the physician, as well as local clinical practice guidelines. For example, in the training cohort, when avoiding missed diagnoses is critical, using a lower threshold such as 0.200 achieves a very high sensitivity of 99.34%, while still reducing unnecessary biopsies by 10.79%. In resource-limited settings or those requiring strict control over the number of biopsies, a threshold of 0.500 reduces unnecessary biopsies by 33.49%, although sensitivity decreases to 91.53%.

## Discussion

In this study, we developed five models to discriminate between benign and malignant thyroid nodules based on ultrasonography radiomics, ACR TI-RADS, and clinical characteristics. The key finding of this study was that the model of the combined clinic-ACR score-Rad score had a better diagnostic efficiency.

According to earlier research, combining ultrasound features such as the ACR TI-RADS lexicon, blood flow, or hypoechoic halo with clinical characteristics such as sex, age, thyrotropin, or nodule diameter slightly improved the precision of the models in differentiating malignant from benign lesions compared to risk classification methods (20, 21). However, another study found no appreciable differences in the aforementioned clinical traits (22). Age, nodule diameter, and ACR TI-RADS score were found in our study to be independent predictors of thyroid cancer in the training group. However, the model combining clinical features with the ACR-score only provides a modest degree of diagnostic effectiveness (AUC=0.784).

The performance gap between the Clin+ACR model (AUC=0.784) and our integrated Clin+ACR+Rad model (AUC=0.958) highlights the limitations of conventional assessment methods and the complementary value of radiomics analysis. This significant improvement (ΔAUC=0.174) can be attributed to several factors. The ACR TI-RADS system, while standardized, relies on subjective visual assessment of a limited set of predefined categorical features, potentially missing subtle variations relevant to malignancy prediction and being subject to inter-observer variability (10). Similarly, clinical features such as age and nodule diameter, though statistically significant, have limited discriminatory power when used alone or combined with ACR-scores. In contrast, radiomics features provide quantitative, objective measurements of nodule characteristics at a level of detail beyond human visual perception, analyzing pixel-level data to quantify subtle aspects of texture, heterogeneity, and morphology that reflect underlying biological properties (11). The significant performance improvement demonstrates that quantitative image analysis captures complementary information not contained in clinical parameters or conventional ultrasound assessments, supporting the value of integrating radiomics into clinical thyroid nodule evaluation.

The ACR TI-RADS can satisfy the original goals of various guidelines, including a reduction in the frequency of unnecessary biopsies and prediction of malignant thyroid nodules. Nevertheless, the clinical application of The ACR guideline is arbitrary (22, 23); malignant lesions can be misclassified if the composition evaluation is inaccurate. Park et al. proved that radiomics dramatically enhances performance and reduces the rate of needless FNAs when paired with ACR recommendations (23). Similarly, Zhang et al. recently developed a radiomics nomogram combining ACR TI-RADS and strain elastography (SE), which demonstrated strong diagnostic performance for thyroid nodules and significantly reduced unnecessary FNA rates (16). Meanwhile, Ren et al. found that a dual-modality radiomics approach based on super-microvascular imaging (SMI) outperformed ACR TI-RADS in classifying thyroid nodules. This approach reduced the unnecessary biopsy rate from 43.4% to 13.9% in the training cohort, and from 45.6% to 18.0% in the validation cohort (24). In another study, Ren et al. developed a dual-modality radiomics nomogram based on B-mode ultrasound and contrast-enhanced ultrasound for ACR TI-RADS 4–5 thyroid nodules. This model showed high accuracy in differentiating benign and malignant TR4–5 nodules and reduced unnecessary FNAB rates (25). These findings are consistent with our results, all demonstrating that models integrating radiomics features with ACR TI-RADS outperforms traditional risk stratification methods. While the research methods differ—our study systematically evaluated five different predictive models to identify the best combination, whereas others focused mainly on specific dual-modality methods—all studies confirm the value and potential of radiomics in thyroid nodule assessment.

While Radiomics gathered internal data from TNs ultrasound pictures at a molecular scale unseen by the human eye to distinguish between malignant and benign TNs, ACR TI-RADS evaluated TNs under macroscopic conditions visible to the unaided eye. Since radiomics and ACR TI-RADS anticipated TNs from two distinct views, benign and malignant, when the two technologies were combined, their complementary impacts improved performance. On this basis, we set up a model of combined clinic-ACR score-Rad score and found better diagnostic efficiency (AUC=0.958); this advantage exists in the test suite as well. This may be because by combining additional data dimensions, we are able to better discriminate between benign and malignant tumors. According to our research, the nomogram of the Clin-ACR-Rad model may be a more practical tool for combining the clinical aspects, ACR-Score, and Rad-Score, and a superior thyroid cancer prognostic model.

Our study has several limitations. First, as a retrospective single-center analysis, selection bias could not be completely avoided, particularly since we only included surgically treated thyroid nodules, limiting the generalizability of our nomogram to patients who did not undergo surgery. To address this important limitation, we are planning a prospective multicenter validation study that will include a more diverse patient population, which will help evaluate the model’s generalizability across different clinical settings. Second, to maintain imaging parameter uniformity, only Philips ultrasound machines were used, suggesting future research should explore various ultrasonic instruments. Third, we did not consider additional dimensional data such as thyroid nodule blood test values, which could enhance prediction accuracy in future models. Furthermore, 98.1% of malignant nodules in our study were papillary thyroid carcinoma, with very few follicular or medullary carcinoma cases. This suggests our model’s performance may primarily apply to papillary thyroid carcinoma prediction, requiring further validation with more diverse samples for other subtypes. Additionally, as our study focused on correlations between ultrasound imaging and pathological results, cytological examination results were not systematically collected. Future research should integrate cytological findings to improve diagnostic accuracy. Lastly, our model lacks external validation from independent medical centers or diverse patient populations, potentially limiting its generalizability to different clinical environments. Future multicenter collaborative studies with prospective designs will further confirm the robustness of the model and enhance the clinical value of our predictive model.

## Conclusions

Overall, the current research offers preliminary support that the model of combined clinic-ACR score-Rad score can be helpful for predicting malignancy in thyroid nodules by examining a retrospective cohort of surgically treated thyroid nodules. The Clin-ACR-Rad nomogram may be a more practical instrument and more accurate prediction model for malignant thyroid nodules.

## Data Availability

The original contributions presented in the study are included in the article/**Supplementary Material**. Further inquiries can be directed to the corresponding author.
