# Supplementary material for: Building radiomics models based on ACR TI-RADS combining clinical features for discriminating benign and malignant thyroid nodules
Source: Front Endocrinol (Lausanne). 2025 Jul 21;16:1486920. doi: 10.3389/fendo.2025.1486920 (PMC12318720; doi:10.3389/fendo.2025.1486920)
Supplement: Supplementary file 1 [file Table1.docx]

Supplementary material. CLEAR Checklist

| No. | Category | Requirement | Completion Status |
| --- | --- | --- | --- |
| Title | | | |
| 1 | Title | Specify the use of radiomics in the title. Consider indicating: type of radiomics technique, imaging modality, key aspects of scan, use of machine learning, external validation, and multi-center design | Completed: Suggested title "Ultrasonographic Radiomics Based on ACR TI-RADS Combined with Clinical Features for Differentiation of Benign and Malignant Thyroid Nodules: A Machine Learning-Based Approach" |
| Abstract | | | |
| 2 | Abstract | Provide a structured abstract including purpose, methods, results, and conclusions. Should specify: baseline characteristics (patients, scans, number of images, etc.), data source, nature of study, segmentation technique, feature extraction technique, dimensionality reduction technique, modeling details, validation technique, testing methods, model performance metrics and uncertainty measurements, number of final feature sets, etc. | Completed: Abstract includes research purpose, methods (323 patients with 329 thyroid nodules, randomly assigned to training and testing cohorts at an 8:2 ratio), results (107 radiomics features extracted, Rad score constructed using LASSO algorithm), and conclusions. Detailed explanation that the study was retrospective, and reported the diagnostic performance of the model (AUC values) |
| Keywords | | | |
| 3 | Keywords | List keywords indicating (e.g., radiomics, texture analysis) and describing the main aspects of radiomics research | Completed: Keywords include radiomics, ACR TI-RADS, thyroid nodules, nomogram, prediction, etc. |
| Introduction | | | |
| 4 | Problem Definition | Define the scientific or clinical problem, summarize relevant literature and knowledge gaps | Completed: Clearly states that thyroid nodules are common diseases of the endocrine system, and determining benign vs. malignant is crucial for patient prognosis and clinical management |
| 5 | Radiomics Rationale | Describe why radiomics methods were considered, the performance and issues of current methods | Completed: The paper explains the limitations of ACR TI-RADS and explains how radiomics can capture subtle tissue features in ultrasound images |
| 6 | Research Purpose | Describe the purpose of the study, focusing on scientific questions | Completed: Clearly lists the specific objectives of the study: (1) extract and select optimal radiomics features; (2) develop and validate prediction models; (3) evaluate the performance of different models; (4) construct a comprehensive nomogram |
| Study Design | | | |
| 7 | CLEAR Guidelines | Indicate that CLEAR checklist was used for reporting | Completed: The paper clearly states adherence to CLEAR guidelines in lines 109-112 |
| 8 | Ethics Issues | Describe ethical issues, ensuring the study was conducted appropriately | Completed: Provides ethics approval information (number 2022-112), statements about patient confidentiality and waiver of informed consent requirements |
| 9 | Sample Size Determination | Describe how sample size was determined | Completed: This is a retrospective study, sample size was based on all patients meeting inclusion and exclusion criteria during the study period (January 2019 to May 2022), rather than a priori sample size calculation. This availability-based sample size determination is appropriate for exploratory radiomics research, and finally included 329 thyroid nodules from 323 patients. |
| 10 | Study Nature | Indicate whether the study is prospective, retrospective, etc. | Completed: Clearly states this is a retrospective study (line 106) |
| 11 | Inclusion/Exclusion Criteria | Define inclusion and exclusion criteria | Completed: Provides clear nodule inclusion and exclusion criteria (lines 117-125) |
| 12 | Technical Flowchart | Provide a technical flowchart | Completed: Paper mentions "Figure 1 describes the flowchart of the radiomics analysis procedure" (lines 190-191) |
| Data | | | |
| 13 | Data Source | Specify data source (private, public, mixed) | Completed: Private institutional data was used |
| 14 | Data Overlap | Indicate if any part of the dataset has been used in previous publications | Completed: No part of the dataset has been used in previous publications |
| 15 | Data Partitioning | Describe the method of partitioning data into training, validation, and test sets | Completed: Random partitioning, 8:2 ratio (training:testing) |
| 16 | Imaging Protocol | Provide imaging protocol and acquisition parameters | Completed: Used Philips iU22 system with 5-12 MHz linear array transducer |
| 17 | Non-Radiomics Variables | Describe data elements used as non-radiomics predictive variables | Completed: Includes clinical data such as age, gender, BMI, medical history, smoking and alcohol consumption status |
| 18 | Reference Standard | Describe the reference standard or outcome measurement that the radiomics method will predict | Completed: Reference standard is pathological results after surgical resection |
| Segmentation | | | |
| 19 | Segmentation Tool | Specify software program or tool used for segmentation | Completed: Mentions the use of ITK-SNAP software (version 3.6.0) with manual segmentation method |
| 20 | Operator Information | Specify the number of operators performing segmentation and their experience | Completed: Performed by two experienced radiologists (with 10 and 7 years of experience respectively) |
| Preprocessing | | | |
| 21 | Preprocessing Tools | Specify software program or tools used for preprocessing | Completed: Image preprocessing was performed using the open-source Python library "Pyradiomics V1.3.0" with described processing steps |
| 22 | Resampling Technique | Specify resampling technique | Completed: B-spline interpolation method was used for image resampling |
| 23 | Discretization Method | Specify discretization method used for handcrafted radiomics feature extraction | Completed: Discretization utilized fixed bin width method with bin width of 25 |
| 24 | Image Type | Provide the image type from which radiomics features were extracted | Completed: Radiomics features were extracted from the original data of B-mode ultrasonography (BMUS) gray-scale images, without using filtered or transformed derivative images |
| Feature Extraction | | | |
| 25 | Extraction Software | Specify software used for radiomics feature extraction | Completed: Used open-source Python library "Pyradiomics V1.3.0" |
| 26 | Feature Classes | Provide radiomics feature classes | Completed: Includes first-order statistical features, gray-level co-occurrence matrix features, gray-level run-length matrix features, etc. (seven classes of features) |
| 27 | Total Number of Features | Specify total number of features per instance | Completed: 107 radiomics features were extracted |
| 28 | Default Parameters | State that remaining parameters were kept at default configuration | Completed: Stated that all other parameters in Pyradiomics V1.3.0 were kept at default configuration |
| Data Preparation | | | |
| 29 | Missing Data | Indicate if missing data existed in the study | Completed: According to inclusion/exclusion criteria, all 329 thyroid nodule samples included in this study had complete clinical data, ultrasound images, and pathological results, with no missing data |
| 30 | Class Imbalance | Indicate class imbalance status | Completed: Among the 329 thyroid nodule samples in this study, 168 (51.1%) were benign nodules and 161 (48.9%) were malignant nodules, with the number of samples in the two categories being nearly balanced |
| 31 | Segmentation Reliability | Describe reliability analysis evaluating the impact of segmentation variation | Completed: Intra- and inter-segmenter consistency assessment was performed |
| 32 | Standardization Technique | Describe standardization technique applied to radiomics feature data | Completed: The radiomics features extracted in this study underwent Z-score standardization before model construction, giving each feature zero mean and unit standard deviation |
| Modeling | | | |
| 33 | Dimensionality Reduction | Specify dimensionality reduction methods used | Completed: Used t-test to identify significant features (p<0.05), Spearman correlation analysis to exclude features with correlation <0.8, and LASSO regression for final feature selection |
| 34 | Modeling Software | Provide the name and version of software program or package used for modeling | Completed: Used R software (version 3.7.0) and SPSS software (version 22.0) for data analysis |
| 35 | Training Process | Describe the training process | Completed: This study used 5-fold cross-validation to optimize model parameters |
| 36 | Confounding Factors | Describe detection methods for potential confounding factors | Completed: This study considered some potential confounding factors in design and implementation. During image acquisition, unified ultrasound equipment and standardized imaging parameters reduced device-related differences. Image quality was standardized during image processing. A blind design was used during segmentation to reduce cognitive bias. Image data was standardized before feature extraction to reduce data distribution differences |
| 37 | Model Selection | Describe the method of selecting the final model | Completed: This study constructed multiple models and compared them to select the optimal model |
| Evaluation | | | |
| 38 | Test Type | Clearly state whether the model was tested internally or externally | Completed: Used internal testing (8:2 split of training and testing sets) |
| 39 | Performance Metrics | Specify performance metrics used to evaluate model predictive ability | Completed: Used area under the ROC curve (AUC), sensitivity, specificity, and other indicators |
| 40 | Uncertainty Assessment | Describe uncertainty assessment | Completed: This study provided calibration curves |
| 41 | Comparison Methods | Specify statistical methods used to compare model performance | Completed: This study evaluated the best predictive model by comparing the diagnostic performance of different combination models. We used the area under the receiver operating characteristic (ROC) curve (AUC) as the main evaluation metric, defining AUC>0.9 as high diagnostic performance, AUC=0.7-0.9 as moderate performance, and AUC=0.5-0.7 as low performance. In addition to AUC, the sensitivity, specificity, positive predictive value (PPV), and negative predictive value (NPV) of each model were calculated to comprehensively evaluate diagnostic efficacy |
| 42 | Method Comparison | Indicate whether comparison with non-radiomics methods was performed | Completed: Compared different combination models of radiomics, clinical, and ACR scores |
| 43 | Interpretability | Describe techniques used to enhance model interpretability | Completed: This study provided a nomogram |
| Results | | | |
| 44 | Baseline Characteristics | Provide baseline demographic and clinical characteristics | Completed: Baseline information provided in Tables 1 and 2 |
| 45 | Flowchart | Provide eligibility criteria flowchart | Completed: Eligibility criteria flowchart provided in Figure 1 |
| 46 | Feature Statistics | Provide statistical information for selected features | Completed: Detailed list of 8 radiomics features and their coefficients |
| 47 | Performance Metrics | Provide performance metrics for training, validation, and testing data | Completed: Provides performance metrics (AUC, sensitivity, specificity, etc.) for different models on training and testing sets |
| 48 | Method Comparison Results | Provide results comparing radiomics methods with non-radiomics or combined methods | Completed: Compared the performance of Rad-score, Clin+Rad, ACR+Rad, Clin+ACR, and Clin+ACR+Rad models |
| Discussion | | | |
| 49 | Summary of Findings | Provide a summary of the work and overview of most important findings | Completed: Summarized the main findings of the study: Clin+ACR+Rad model has better diagnostic efficiency |
| 50 | Related Work | Provide the most important and relevant previous work | Completed: Reviewed previous related studies, pointing out the innovations of this study |
| 51 | Practical Significance | Summarize the practical significance of the results | Completed: Discussed the advantages of the Clin-ACR-Rad nomogram as a practical tool and predictive model |
| 52 | Strengths and Limitations | Clearly state the strengths and limitations of the current work | Completed: Discussed the limitations of the study, including selection bias in single-center retrospective studies, etc. |
