# Supplementary material for: Building radiomics models based on ACR TI-RADS combining clinical features for discriminating benign and malignant thyroid nodules
Source: Front Endocrinol (Lausanne). 2025 Jul 21;16:1486920. doi: 10.3389/fendo.2025.1486920 (PMC12318720; doi:10.3389/fendo.2025.1486920)
Supplement: Supplementary file 2 [file Table2.docx]

Supplementary Table. Comparison of Unnecessary Biopsy Rates Between the Combined Model and ACR TI-RADS at Various Thresholds.

| Cohort | Threshold | Sensitivity (%) | Specificity (%) | Biopsies Recommended | Unnecessary Biopsies | Unnecessary Biopsy Rate (%) | Missed Malignancies |
| --- | --- | --- | --- | --- | --- | --- | --- |
| Training Cohort (n=263) |  |  |  |  |  |  |  |
| ACR TI-RADS | - | 100.00 | 0.00 | 198 | 93 | 46.97 | 0 |
| Clin+ACR+Rad | 0.200 | 99.34 | 65.43 | 152 | 55 | 36.18 | 1 |
|  | 0.300 | 98.72 | 79.25 | 124 | 37 | 29.84 | 2 |
|  | 0.386* | 98.43 | 89.24 | 127 | 28 | 22.05 | 2 |
|  | 0.400 | 96.44 | 91.54 | 112 | 22 | 19.64 | 4 |
|  | 0.500 | 91.53 | 95.82 | 89 | 12 | 13.48 | 10 |
| Test Cohort (n=66) |  |  |  |  |  |  |  |
| ACR TI-RADS | - | 100.00 | 0.00 | 48 | 22 | 45.83 | 0 |
| Clin+ACR+Rad | 0.200 | 97.18 | 63.69 | 37 | 13 | 35.14 | 1 |
|  | 0.300 | 95.75 | 75.86 | 32 | 9 | 28.13 | 1 |
|  | 0.386* | 93.90 | 79.64 | 38 | 8 | 21.05 | 2 |
|  | 0.400 | 92.95 | 82.45 | 35 | 6 | 17.14 | 2 |
|  | 0.500 | 88.67 | 89.14 | 26 | 3 | 11.54 | 4 |

*Optimal threshold based on Youden's index.
